# Pre-treatment of melatonin enhances the seed germination responses and physiological mechanisms of soybean (*Glycine max* L.) under abiotic stresses

**DOI:** 10.3389/fpls.2023.1149873

**Published:** 2023-03-06

**Authors:** Samrah Afzal Awan, Imran Khan, Qi Wang, Jing Gao, Xianming Tan, Feng Yang

**Affiliations:** ^1^College of Agronomy, Sichuan Agricultural University, Chengdu, China; ^2^Sichuan Engineering Research Center for Crop Strip Intercropping System, Chengdu, China; ^3^State Key Laboratory of Grassland Agro-Ecosystems, Ministry of Agriculture, College of Pastoral Agriculture Science and Technology, Lanzhou University, Lanzhou, China

**Keywords:** soybean, melatonin, germination, oxidative damage, antioxidant enzymes

## Abstract

The germination of soybean (*Glycine max* L.) seeds is critically affected by abiotic stresses which resulting in decreasing crop growth and yield. However; little is known about the physiological mechanisms of germination and the potential role of melatonin on soybean seed germination under drought, salt, cold, and heat stresses. Therefore, the current study investigated the possible effects of melatonin to enhance germination indices and other physiological attributes by alleviating the harmful impacts of these stresses during germination. Seeds of soybean were pre-treated (seed priming) with melatonin at MT1 (20 μmol L^-1^), MT2 (50 μmol L^-1^), MT3 (100 μmol L^-1^), MT4 (200 μmol L^-1^), and MT5 (300 μmol L^-1^) and exposed to the four stresses (drought at PEG 15%, salt at 150mM, cold at 10 °C, and heat at 30 °C) . It was noted that MT1 (20 μmol L^-1^), MT2 (50 μmol L^-1^), and MT3 (100 μmol L^-1^) remarkably improved the germination potential, germination rate, radical length, and biomass under given stresses. Furthermore, MT1, MT2, and MT3 progressively increased the proline to minimize the impact of drought, salt, cold, and heat stresses. In addition, all stresses significantly induced oxidative damage however, salt (150 mM NaCl) and heat (30 °C) stresses highly increased the malondialdehyde content (MDA) and hydrogen peroxide (H2O2) as compared to drought (PEG 15%) and cold (10 °C) stresses. Moreover, MT2 and MT3 significantly enhanced the activities of antioxidant enzymes such as superoxide dismutase (SOD), catalase (CAT), peroxidase (POD), and ascorbate peroxidase (APX) to reduce the oxidative damage in soybean seeds during the germination. Overall, melatonin at 50 μmol L^-1^ and 100 μmol L^-1^ considerably mitigated the harmful impacts of drought, salt, cold, and heat stress by enhancing germination and other physiological mechanisms of soybean. This study could provide bases to enhance the melatonin-mediated tolerance of soybean and other related crops at early growth stages when exposed to abiotic stresses.

## Introduction

Seed germination starts as dry seed absorbs water and ends as radical protrudes from the seed coat, and it is a crucial stage of a plant’s life cycle (Xiao et al., 2019). A series of metabolic, cellular, and molecular events are usually involved in this complex process for the successful establishment of crops at early growth stages (Zhang et al., 2020). The seed germination stage is significantly influenced by external environmental factors and is very sensitive to abiotic stresses such as drought, salt, and low and high temperatures. Therefore, this stage makes a strong interaction between growth and the final yield of crops which refers to economic and ecological importance (Weitbrecht et al., 2011). Abiotic stresses cause irregular seedling emergence and lead to decrease in number of plants and final crop production (Okçu et al., 2005). Changes in the germination indicators including germination index (GI), germination potential (GP), and germination rate (GR) could be considered for the evaluation of abiotic stress tolerance during seed germination (Zhang et al., 2020). It has been reported that mechanical strength provided by seed coat increases under stressful condition which directly inhibits seed germination (Debeaujon et al., 2000). Abiotic stress condition induces oxidative damage by increasing the excessive production of reactive oxygen species (ROS) in the plant cell, resulting in cell death. However, a complex antioxidant defense system efficiently regulates the oxidative profile by enhancing the activities of antioxidant enzymes such as superoxide dismutase (SOD), catalase (CAT), peroxidase (POD), and ascorbate peroxidase (APX), and reduces the impacts of stresses (Xiao et al., 2019). Therefore, it is crucial to reduce the impacts of abiotic stresses on seed germination for better seedling growth and crop yield.

Different conventional methods have been applied to lessen the deleterious effects of abiotic stresses on seed germination and radical emergence (Seleiman et al., 2021). Among them, seed pre-treatment with biological substances, biomolecules, or phytohormones could be important to enhance seed germination and tolerance to abiotic stresses (Awan et al., 2021). Melatonin is a biological molecule produced by L-tryptophan and performs a variety of physiological functions in plants (Imran et al., 2021). Recently, melatonin is being widely used by different researchers to improve growth, productivity, and tolerance of plants under various types of stresses and found beneficial for plants under normal and stressful conditions (Wang et al., 2018). The production of endogenous melatonin is not enough and cannot significantly reduce the impact of abiotic stresses due to high degree of oxidative damage caused by over production of ROS (Fu et al., 2017). Melatonin effectively regulates seed germination, growth and development, and antioxidant profile in plants. Furthermore, the impact of melatonin on seed germination is dose-dependent, however; its pre-treatment usually promotes seed germination and seedling emergence even under stressful conditions (Xiao et al., 2019). Pieces of evidence reported that exogenous melatonin reduced the malondialdehyde (MDA) content, H_2_O_2_, oxygen radical, and electrolyte leakage in the seedlings under stressful conditions (Bai et al., 2020; Wei et al., 2022). Therefore, the use of melatonin as a seed germination-promoting agent under stressful conditions could be crucial for crop growth and development.

Soybean (*Glycine max* [L.] Merr.) is one of the most important economical grain crops cultivated for oil and protein worldwide (Papadaki et al., 2019; Zhao et al., 2021). Soybean roots have good nitrogen fixation ability, which can reduce the excessive use of fertilizer and is beneficial to the sustainable development of the environment (Meena et al., 2018). Soybeans are used as a major source of food and for feeding and have high economic value, high content of protein and oil, minerals, nutrients, and vitamins (Yang et al., 2018b). Besides, soybean is also being used in intercropping with maize and other crops to achieve the goal of safe and enough production within the limited space (Yang et al., 2014; Yang et al., 2018a) due to the increasing world population. The worldwide production of soybean was reported 311.1 million tonnes in 2020 and estimated to reach 371.3 million tonnes in 2030, which indicated 1.8% more growth rate as compared to 2010-2020 (Siamabele, 2021). According to a conducted survey, soybean farming increased the agriculture business income by 37.77% and household income by 18.87% in Indonesia (Roessali et al., 2019). In 2017, the total production of soybean was 14.3 million tonnes in China and due to less land available for soybean cultivation, China imported soybean about 95.5 million tonnes to fulfill the need of soybean (Guo et al., 2021). This suggested that soybean farming (increasing growth rate) could improve its production that can increase the agriculture business income and household’s income in China and could be important to economy of China. In addition, the soybean intercropping with other crops usually creates a low-light environment that could be a challenge to successful growth and sufficient production of soybean because it also refers to a stressful environment (Yang et al., 2017; Yang et al., 2020). However, soybean production hardly meets the needs of the increasing global population. At the same time, unfavorable environmental settings and a decrease in the germination potential in terms of low germination rate due to abiotic stresses negatively affect its early growth which leads to fatal disorders (Zhang et al., 2020). Soybean is highly sensitive to drought, salt, and heat stresses that inhibited seed germination, vegetative growth, and physiological, and biochemical attributes (Zhang et al., 2020; Imran et al., 2021). Abiotic stresses generally lead to reduce water supply to seed or germinating seeds which results in slow metabolic processes and inhibits or prolongs seed germination that negatively affects its later growth stages (Bai et al., 2020). The protective role of melatonin pre-treatment on soybean seed germination and seedling growth under combination of abiotic stresses (drought, salt, cold, and heat) is still unclear that needs to be investigated for clear understandings.

Therefore, the present study was designed to investigate the potential role of melatonin to mitigate the impact of abiotic stress and promote seed germination of soybean. Moreover, how melatonin can regulate the different physiological mechanisms during seed germination of soybean under different types of stresses is also crucial to be explored for future research work. This is because, little is known about the pre-treatment (seed priming) of melatonin on soybean seed germination, its physiological profile, and antioxidant enzyme activities under drought, salt, cold, and heat stresses. Thus, the main objectives of the current study were to investigate the role of pre-treatment of melatonin on soybean seed germination indices, seedling growth, degree of oxidative damage, activities of antioxidant enzyme activities, and range of tolerance under drought, salt, cold, and heat stresses

## Materials and methods

### Plat materials, treatments, and growth conditions

The seeds of soybean (*Glycine max* [L.] Merr.) cultivar Nandou-12 (ND-12) were used in this experiment and obtained from Nanchong Academy of Agricultural Sciences, Sichuan Province, China. Healthy and uniform size seeds of the soybean cultivar “Nandou 12” were surface sterilized by following the method given by (Awan et al., 2022). After that seeds were air-dried and subjected to different melatonin (MT) treatments. Moreover, the melatonin (molecular weight: 232.28) was purchased from Sigma Aldrich with >99% purity. To make a stock solution of melatonin, the amount of melatonin 0.232g powder was weighed and dissolved in an appropriate amount of anhydrous ethanol. Later, deionized water was added and made the final volume of the solution up to 100 mL which gave 10,000 μmol L^-1^. Further dilutions were carried out to prepare different concentrations of melatonin as CK, MT1 (20 μmol L^-1^), MT2 (50 μmol L^-1^), MT3 (100 μmol L^-1^), MT4 (200 μmol L^-1^), and MT5 (300 μmol L^-1^), and seeds were dipped in the prepared concentrations for 12 hours (pre-treatment). After that seeds were air-dried at room temperature for 10 min and cultivated in petri plates having moistened blotting paper. A total of 100 seeds were cultivated for each treatment and placed petri plates in the growth chambers under growing conditions of 8 hours at night and 12 hours a day.

### Drought (PEG), salt, cold, and heat stress treatments

All the seed pre-treated with melatonin was separated into four groups and exposed to these four stresses in petri plates. After immediate cultivation of soybean seeds into petri plates, the 15% PEG-6000 by dissolving in distilled water, and 150 mM NaCl by dissolving in distilled water up to final concentration (150mM), cold (10°C), and heat (30°C) were given to seeds. For PEG and NaCl, 10 mL of solution was applied after every 24 hours to each petri plate except for the control (CK). The germination of seeds was observed and noted daily and after 7 days, the related parameters were measured.

### Germination indices

Germination indices including germination potential and germination rate of soybean seeds were measured according to the method given by (Cao et al., 2019; Chen et al., 2020) under all given treatments by using the following formulae;


Germination potential (%) = number of seeds germinated at 3 days/ total no. of seeds × 100

Germination rate (%) = number of germinated seeds at 7 days/ total no. of seeds × 100

### Proline content and Electrolyte Leakage

Proline content was measured according to the method given by (Bates et al., 1973) with slight modifications. The fresh radical sample (0.5 g) from each treatment was homogenized with 3 mL of 3% (w/v) sulfosalicylic acid and centrifuged. The supernatant was collected and 2mL of each glacial acetic acid and ninhydrin was added to the supernatant and heated for 30 min at 100˚C in a water bath. Later, the mixture was placed in an ice bath to stop the reaction. After cooling, the mixture was again centrifuged at 10,000 rpm for 5 min and absorbance of the mixture was measured at 520nm using a spectrophotometer. The proline content was expressed as μmol g^-1^ FW. Moreover, the electrolyte leakage (EL) of fresh sample material was determined according to the method described by (Chen et al., 2020).

### Determination of malondialdehyde and hydrogen peroxide

The MDA and H_2_O_2_ were determined according to the instructions of the kits (Solarbio, Beijing) as reported by (Gu et al., 2018; Zhao et al., 2018). In brief, 0.5 g of sample material was ground with liquid nitrogen and extraction buffer was added to it. Later, the reaction mixture was centrifuged at 12,000 g for 10 min and the supernatant was collected. Finally, the MDA and H_2_O_2_ were determined as per manufacturer instructions.

### Measurements of antioxidant enzymes activities

The activities of antioxidant enzymes including superoxide dismutase (SOD), catalase (CAT), peroxidase (POD), and ascorbate peroxidase (APX) were measured by following the instructions of antioxidant enzyme assay kits (Solarbio) as reported by (Zhao et al., 2018). In short, 0.5g of the sample was ground in liquid nitrogen and extracted through an extraction buffer. Later, the extraction mixture was centrifuged at 12, 000 g for 10 min, and the supernatant was collected. The activities of SOD, CAT, POD, and APX were measured by using a spectrophotometer.

### Statistical analyses

All the data were analyzed by one-way analyses of variance (ANOVA) using statistics 8.1. Data are the mean of triplicate per treatment with standard deviation (mean ± SD). The statistical differences among different treatments were achieved by the least significance difference (LSD) test. The level of significance was considered at *p <* 0.05. The graphical figures of the present study were generated by using Microsoft excel. Principal component analysis (PCA) among various measured variables of soybean under different types of stresses was achieved using “past” software.

## Results

**Figure 1:**
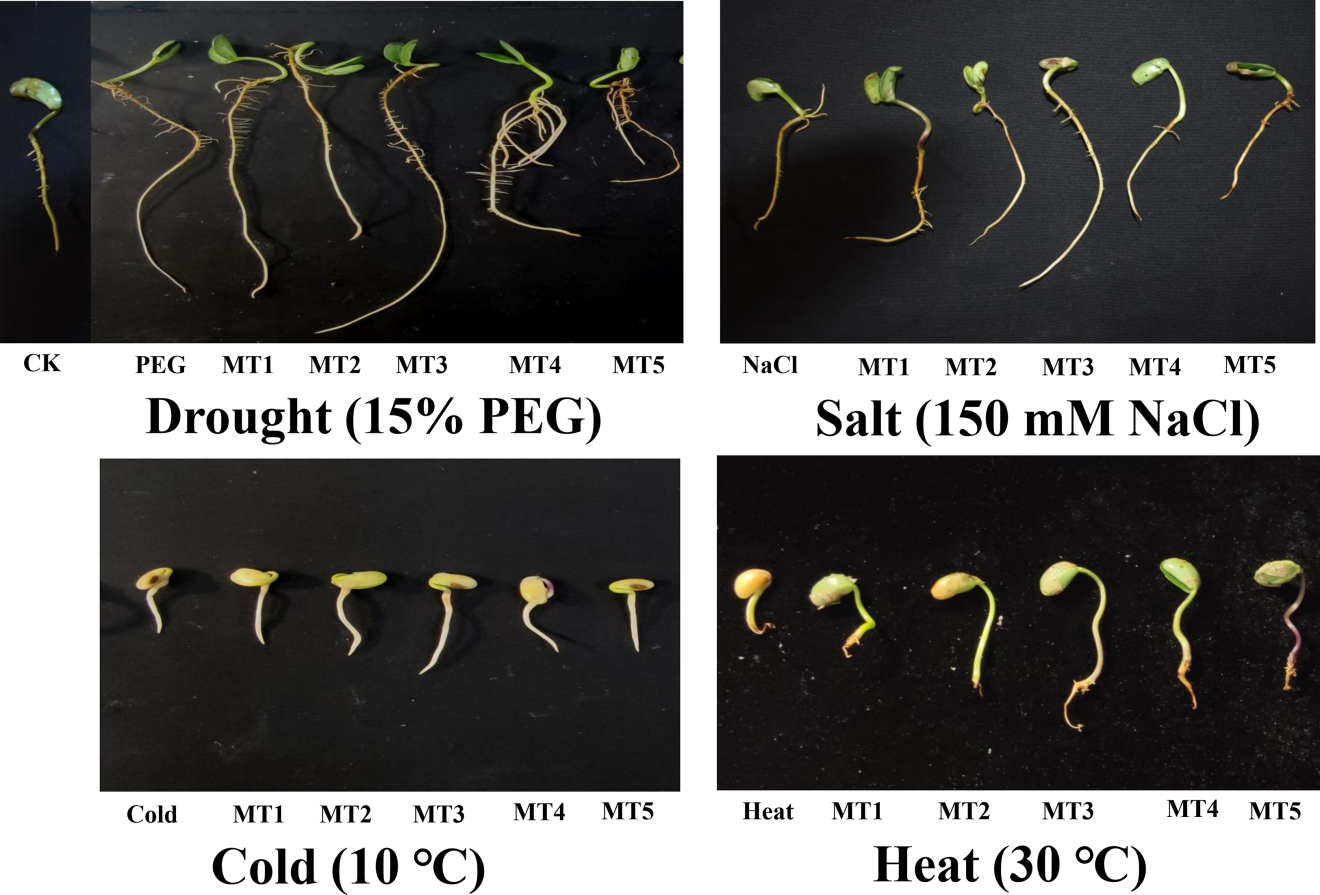
Effect of different concentrations of melatonin on the morphological representation of soybean germination PEG (15%), NaCl (150mM), Cold (10 °C), and Heat (30°C).

The results of the present study depicted that PEG, NaCl, Cold, and Heat stresses remarkably declined the germination indices including germination potential, germination rate, radical lengths, and fresh and dry weights of germinated soybean seeds (**Table 1**). However, different concentrations of melatonin differentially improved the morphology and germination indices by reducing the harmful effects of these four stresses (**Figure 1**). Furthermore, PEG, NaCl, Cold, and Heat stress reduced the germination potential by 33%, 36%, 36%, and 41% and germination rate by 30%, 27%, 29%, and 36% over the respective controls. The PEG stress differently influenced the radical length and fresh and dry weight of soybean seeds during germination on exposure to different concentrations of melatonin. In addition, NaCl, Cold, and Heat stress reduced the radical length by 19%, 71%, and 71% and dry weight by 22%, 49%, and 65% as compared to the control, respectively.

**Table 1:** Effect of different concentrations of melatonin on germination indices, radical length, and biomass of germinated soybean seeds under PEG, NaCl, Cold, and Heat stress.

Treatments		Germination Potential (%)	Germination Rate (%)	Radical Length (cm)	Fresh Weight (g)	Dry Weight (g)
**PEG-stress**	CK	86.0 ± 3.4a	86.0 ± 3.4a	13.5 ± 0.20a	0.71 ± 0.008c	0.042 ± 0.0022b
	stress	57.3 ± 2.3d	60.0 ± 2.6c	20.6 ± 0.31f	0.73 ± 0.012c	0.035 ± 0.0013c
	MT1 (20µM)	71.7 ± 2.6bc	75.0 ± 1.7b	18.3 ± 0.16e	0.72 ± 0.005c	0.041 ± 0.0011b
	MT2 (50µM)	78.3 ± 2.0ab	83.3 ± 2.3a	15.4 ± 0.17d	0.77 ± 0.012b	0.043 ± 0.0015b
	MT3 (100µM)	80.6 ± 2.9a	85.3 ± 2.4a	21.9 ± 0.28b	0.82 ± 0.008a	0.049 ± 0.0011a
	MT4 (200µM)	70.1 ± 2.1bc	70.6 ± 2.6b	15.3 ± 0.24c	0.78 ± 0.006b	0.034 ± 0.0015c
	MT5 (300µM)	65.0 ± 3.0cd	74.6 ± 2.1b	11.4 ± 0.14c	0.72 ± 0.006c	0.032 ± 0.0018c
**NaCl-stress**	CK	86.0 ± 3.4a	86.0 ± 3.4a	13.5 ± 0.20a	0.71 ± 0.008c	0.042 ± 0.0022ab
	stress	55.0 ± 3.4f	62.0 ± 1.5d	10.9 ± 0.20f	0.64 ± 0.008b	0.032 ± 0.0017c
	MT1 (20µM)	71.3 ± 2.0cd	76.3 ± 2.1b	14.5 ± 0.20de	0.64 ± 0.005c	0.036 ± 0.0018bc
	MT2 (50µM)	82.2 ± 2.0ab	87.0 ± 1.0a	13.8 ± 0.03cd	0.68 ± 0.003c	0.033 ± 0.0022c
	MT3 (100µM)	76.0 ± 2.6bc	77.6 ± 2.4b	14.8 ± 0.20b	0.74 ± 0.005b	0.044 ± 0.0019a
	MT4 (200µM)	65.6 ± 1.4de	71.6 ± 2.0bc	12.6 ± 0.12c	0.59 ± 0.011a	0.035 ± 0.0016c
	MT5 (300µM)	63 ± 2.0e	68.0 ± 1.7cd	10.7 ± 0.21ef	0.49 ± 0.012d	0.033 ± 0.0018c
**Cold-stress**	CK	86.0 ± 3.4a	86.0 ± 3.4a	13.5 ± 0.20b	0.71 ± 0.008e	0.042 ± 0.0022a
	stress	54.6 ± 2.4e	60.3 ± 2.0e	3.9 ± 0.08d	0.38 ± 0.014a	0.021 ± 0.0016e
	MT1 (20µM)	65.0 ± 1.7cd	71.6 ± 2.0c	4.4 ± 0.06a	0.42 ± 0.005f	0.025 ± 0.0011cd
	MT2 (50µM)	70.3 ± 2.9bc	72.6 ± 2.3bc	4.7 ± 0.05b	0.47 ± 0.011e	0.021 ± 0.0014de
	MT3 (100µM)	77.0 ± 2.0b	79.3 ± 2.6ab	5.1 ± 0.10a	0.52 ± 0.008cd	0.032 ± 0.0015b
	MT4 (200µM)	63.0 ± 2.8cd	64.6 ± 1.7de	4.7 ± 0.03c	0.47 ± 0.006b	0.027 ± 0.0010c
	MT5 (300µM)	60.7 ± 2.1de	69.6 ± 1.2cd	4.2 ± 0.06d	0.44 ± 0.008c	0.023 ± 0.0010cde
**Heat-stress**	CK	86.0 ± 3.4a	86.0 ± 3.4a	13.5 ± 0.20e	0.71 ± 0.008a	0.042 ± 0.0022a
	stress	50.3 ± 1.8d	54.6 ± 1.4d	3.9 ± 0.11b	0.29 ± 0.017e	0.014 ± 0.0017e
	MT1 (20µM)	59.3 ± 1.8c	64.0 ± 1.0c	5.1 ± 0.12c	0.32 ± 0.006e	0.018 ± 0.0011de
	MT2 (50µM)	72.3 ± 2.1b	75.0 ± 1.5b	6.2 ± 0.21d	0.39 ± 0.008cd	0.021 ± 0.0029cd
	MT3 (100µM)	74.0 ± 3.0b	81.6 ± 1.4a	8.1 ± 0.15a	0.48 ± 0.005b	0.030 ± 0.0012b
	MT4 (200µM)	63.0 ± 1.5c	66.0 ± 1.5c	7.3 ± 0.08d	0.40 ± 0.008c	0.023 ± 0.0012cd
	MT5 (300µM)	70.3 ± 2.3b	73.6 ± 2.3b	7.0 ± 0.12f	0.37 ± 0.008d	0.024 ± 0.0020c

Values are the means ± SD (n=3). Different letters show a statistical significance level at *p<0.05*. Here, µM is indicating (µmole L^-1^).

In addition, the combined application of melatonin with PEG at MT1, MT2, MT3, MT4, and MT5 increased the germination potential by 25%, 36%, 40%, 23%, and 13%, germination rate by 25%, 38%, 42%, 17%, and 24%, over the PEG, respectively. The applied concentrations of melatonin showed a progressive role under NaCl, Cold, and Heat stresses. Moreover, the MT1, MT2, MT3, MT4, and MT5 improved the germination potential by 29%, 49%, 38%, 19%, and 14%, germination rate by 23%, 40%, 25%, 15%, and 9%, over the NaCl, respectively. Similarly, MT1, MT2, MT3, MT4, and MT5 enhanced the germination potential by 19%, 28%, 40%, 15%, and 10%, germination rate by 19%, 20%, 31%, 7%, and 15%, over the Cold, respectively. In the same context, MT1, MT2, MT3, MT4, and MT5 increased the germination potential by 17%, 43%, 47%, 25%, and 39%, germination rate by 17%, 37%, 49%, 20%, and 34%, over the Heat, respectively (**Table 1**). In addition, the seed priming at different concentrations of melatonin differentially impacted the fresh and dry biomass of germinated soybean seeds under drought, salt, cold, and heat stresses. However, the optimized concentrations of melatonin significantly improved the fresh and dry biomass of germinated soybean seeds as shown in **Table 1**.

Among all treated concentrations of melatonin, the 20μmol L^-1^ (MT1), 50μmol L^-1^ (MT2), and 100μmol L^-1^ (MT3) showed significant impacts on morphology and germination indices of soybean seeds as compared to other concentrations under PEG, NaCl, Cold, and Heat stresses. Therefore, we selected these three concentrations of melatonin to carry out further analyses.

**Figure 2:**
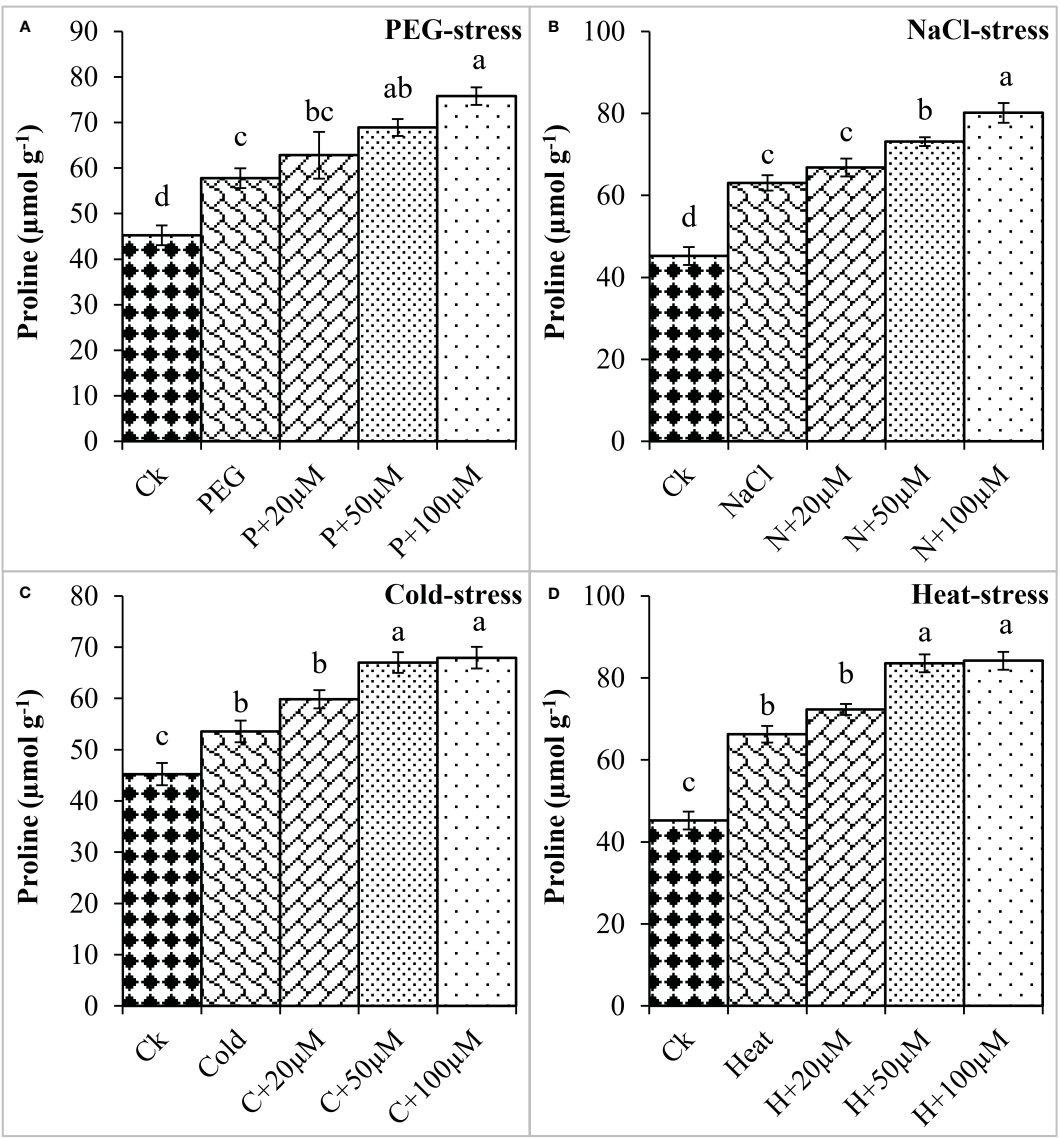
Effect of different concentrations of melatonin on the activity of Proline content under PEG **(A)**, NaCl **(B)**, Cold **(C)**, and Heat **(D)**. Values are the means ± SD (n=3). Different letters on the bars show a statistical significance level at *p<0.05*. Here, µM is indicating (µmole L^-1^).

**Figure 3:**
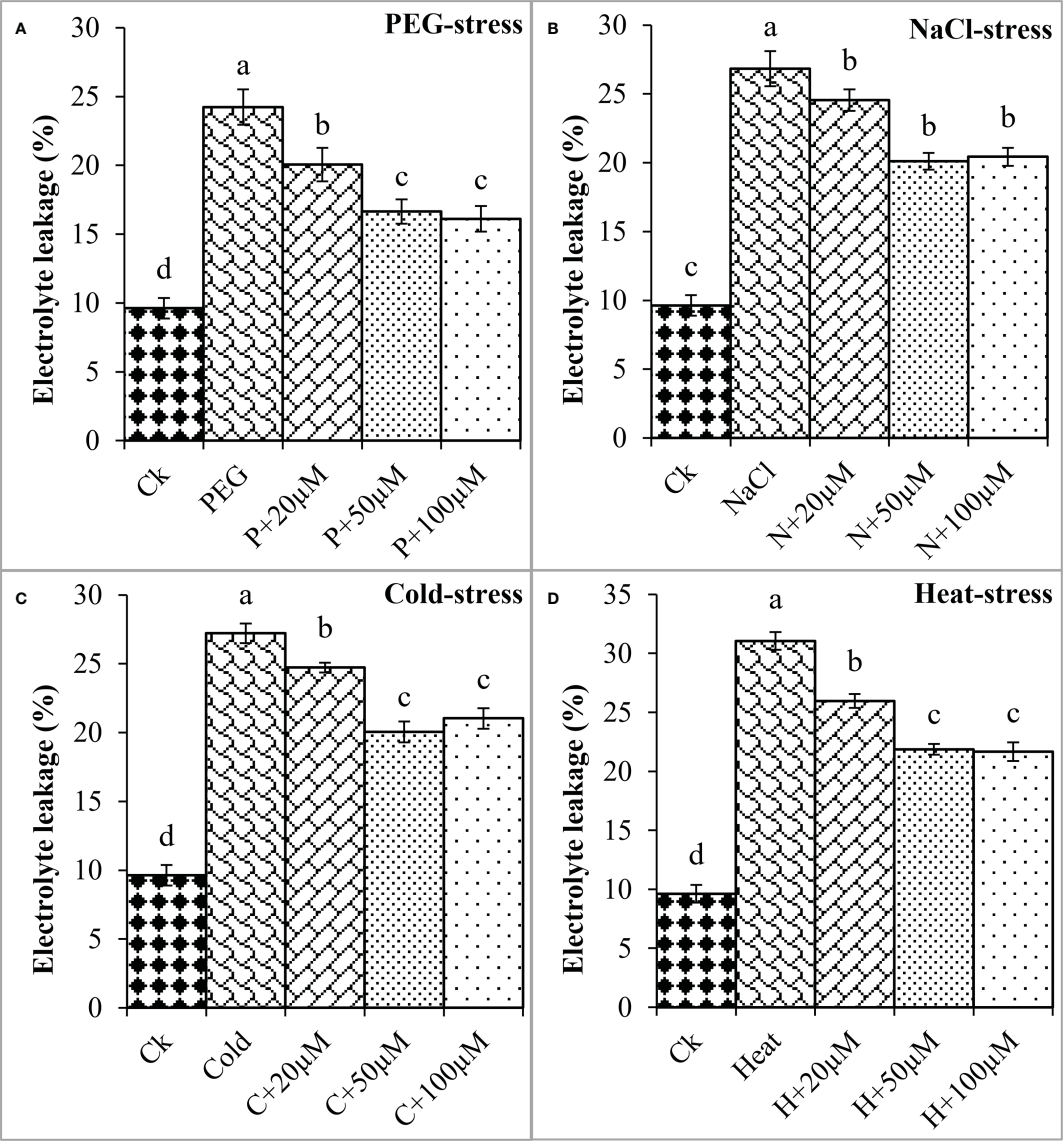
Effect of different concentrations of melatonin on the activity of Electrolyte leakage (EL) under PEG **(A)**, NaCl **(B)**, Cold **(C)**, and Heat **(D)**. Values are the means ± SD (n=3). Different letters on the bars show a statistical significance level at *p<0.05*. Here, µM is indicating (µmole L^-1^).

Proline is an important osmoprotectant that facilitates plants to withstand stressful conditions (Bai et al., 2020). In the present study, PEG, NaCl, Cold, and Heat stress increased the proline content by 27%, 39%, 18%, and 46% as compared to the respective control (**Figure 2**). However, melatonin at 20μmol L^-1^, 50μmol L^-1^, and 100μmol L^-1^ progressively improved the proline content under all these stresses and maximum enhancement was noticed at 50 μmol L^-1^ and 100 μmol L^-1^. Melatonin at 20 μmol L^-1^, 50 μmol L^-1^ and 100 μmol L^-1^ improved the proline by 8%, 19%, and 31% over the PEG (**Figure 2A**), 6%, 16%, and 27% over the NaCl (**Figure 2B**), 11%, 25% and 26% over the Cold (**Figure 2C**), 9%, 26% and 27% over the Heat (**Figure 2D**), respectively. On the other hand, electrolyte leakage (EL) was found to be increased due to PEG, NaCl, Cold, and Heat stress whereas, the application of melatonin reduced EL under these stresses. The EL was found to increase by PEG (151%), NaCl (178%), Cold (182%), and Heat (222%) over the respective controls (**Figure 3**). Furthermore, 20 μmol L^-1^, 50 μmol L^-1^, and 100 μmol L^-1^ decreased EL by 17%, 31%, and 33% over PEG, 8%, 25%, and 23% over NaCl, 9%, 26%, and 22% over Cold, and 16%, 29%, and 30% over Heat stress, respectively. Overall, melatonin at 50 μmol L^-1^, and 100 μmol L^-1^ remarkably improved the proline and reduced the EL under different types of abiotic stresses.

**Figure 4:**
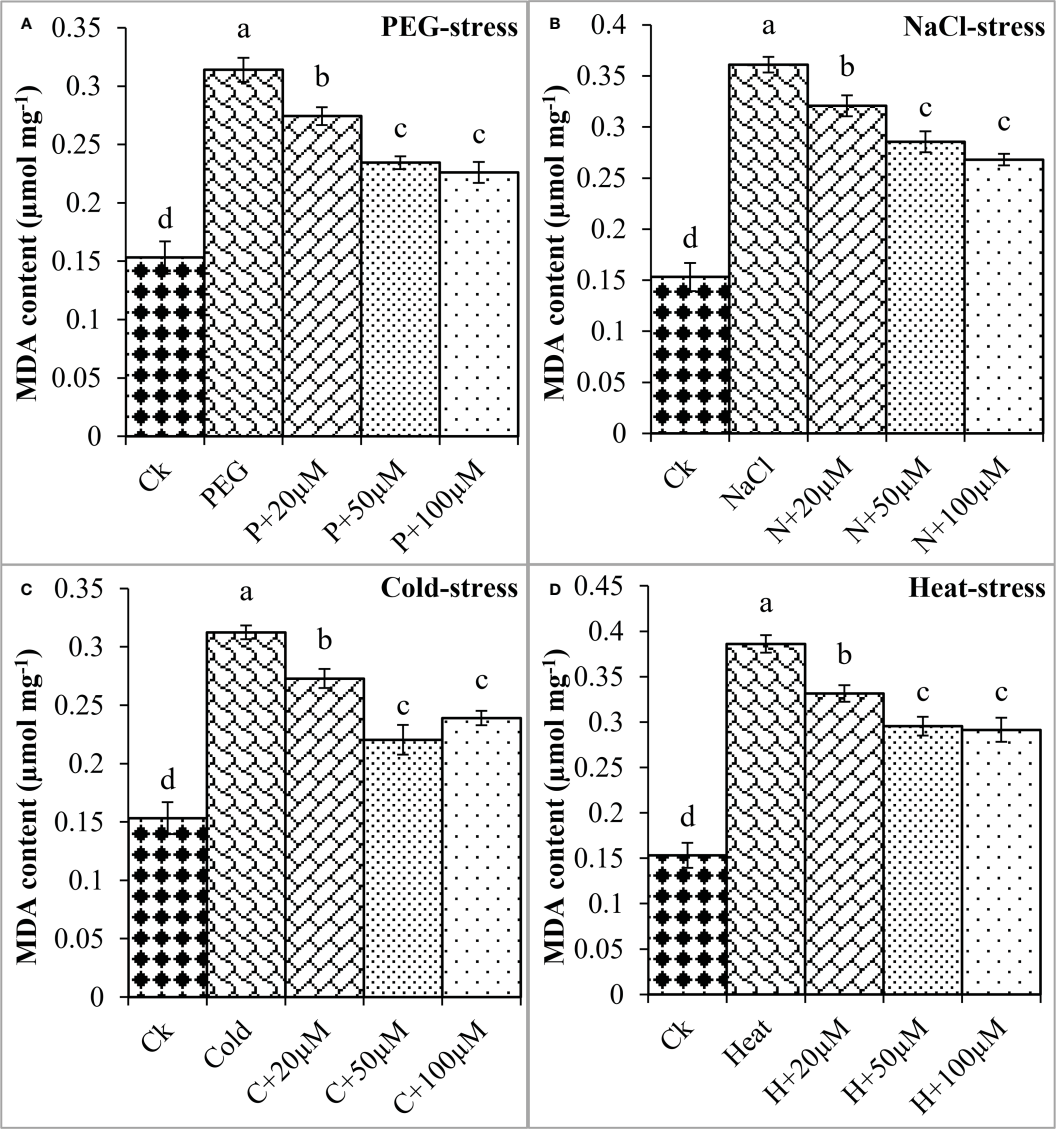
Effect of different concentrations of melatonin on the activity of MDA content under PEG **(A)**, NaCl **(B)**, Cold **(C)**, and Heat **(D)**. Values are the means ± SD (n=3). Different letters on the bars show a statistical significance level at *p<0.05*. Here, µM is indicating (µmole L^-1^).

**Figure 5:**
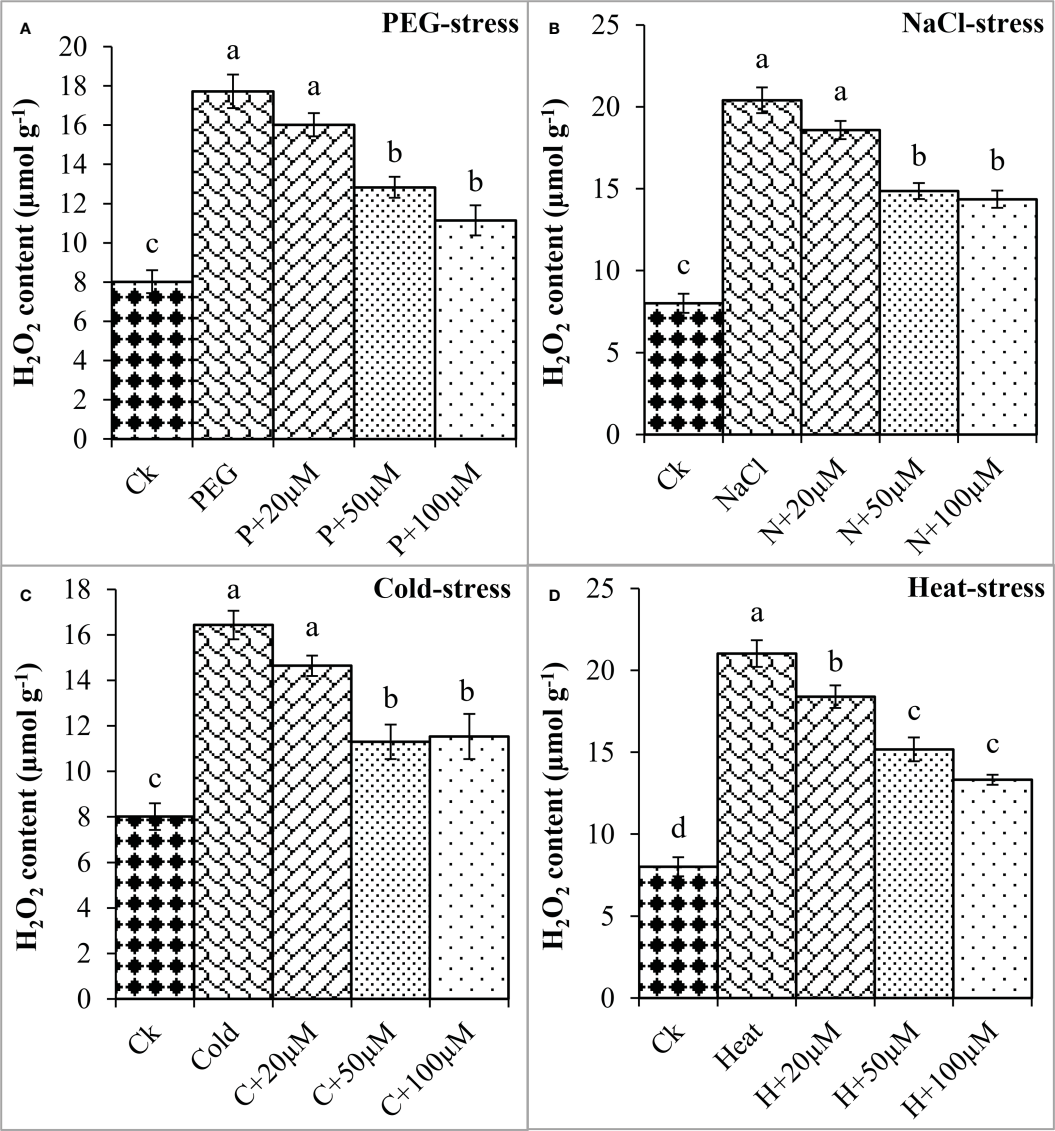
Effect of different concentrations of melatonin on the activity of H_2_O_2_ under PEG **(A)**, NaCl **(B)**, Cold **(C)**, and Heat **(D)**. Values are the means ± SD (n=3). Different letters on the bars show a statistical significance level at *p<0.05*. Here, µM is indicating (µmole L^-1^).

Moreover, oxidative damage in terms of MDA and H_2_O_2_ was found increased due to PEG, NaCl, Cold, and Heat however, melatonin at different concentrations significantly reduced MDA and H_2_O_2_ under these stresses (**Figures 4**, **5**). In the present study, PEG, NaCl, Cold, and Heat stress increased the MDA content by 105%, 135%, 103%, and 152% as compared to the respective control (**Figure 4**). However, melatonin at 20 μmol L^-1^, 50 μmol L^-1^, and 100 μmol L^-1^ linearly decreased the MDA content under all these stresses. Melatonin at 20 μmol L^-1^, 50 μmol L^-1^ and 100 μmol L^-1^ reduced MDA by 13%, 25%, and 28% over the PEG (**Figure 4A**), 11%, 20%, and 25% over the NaCl (**Figure 4B**), 12% 29% and 23% over the Cold (**Figure 4C**), 14%, 23% and 24% over the Heat (**Figure 4D**), respectively. On the other hand, H_2_O_2_ was also increased due to PEG, NaCl, Cold, and Heat stresses by 121%, 154%, 105%, and 162% over their respective controls. Furthermore, 20 μmol L^-1^, 50 μmol L^-1^, and 100 μmol L^-1^ decreased H_2_O_2_ by 9%, 27%, and 37% over PEG, 8%, 27%, and 29% over NaCl, 10%, 31%, and 29% over Cold, and 12%, 27%, and 36% over Heat stress respectively (**Figure 5**). Overall, melatonin at 50µM, and 100µM remarkably reduced the MDA and H_2_O_2_ (oxidative damage) under different types of abiotic stresses.

**Figure 6:**
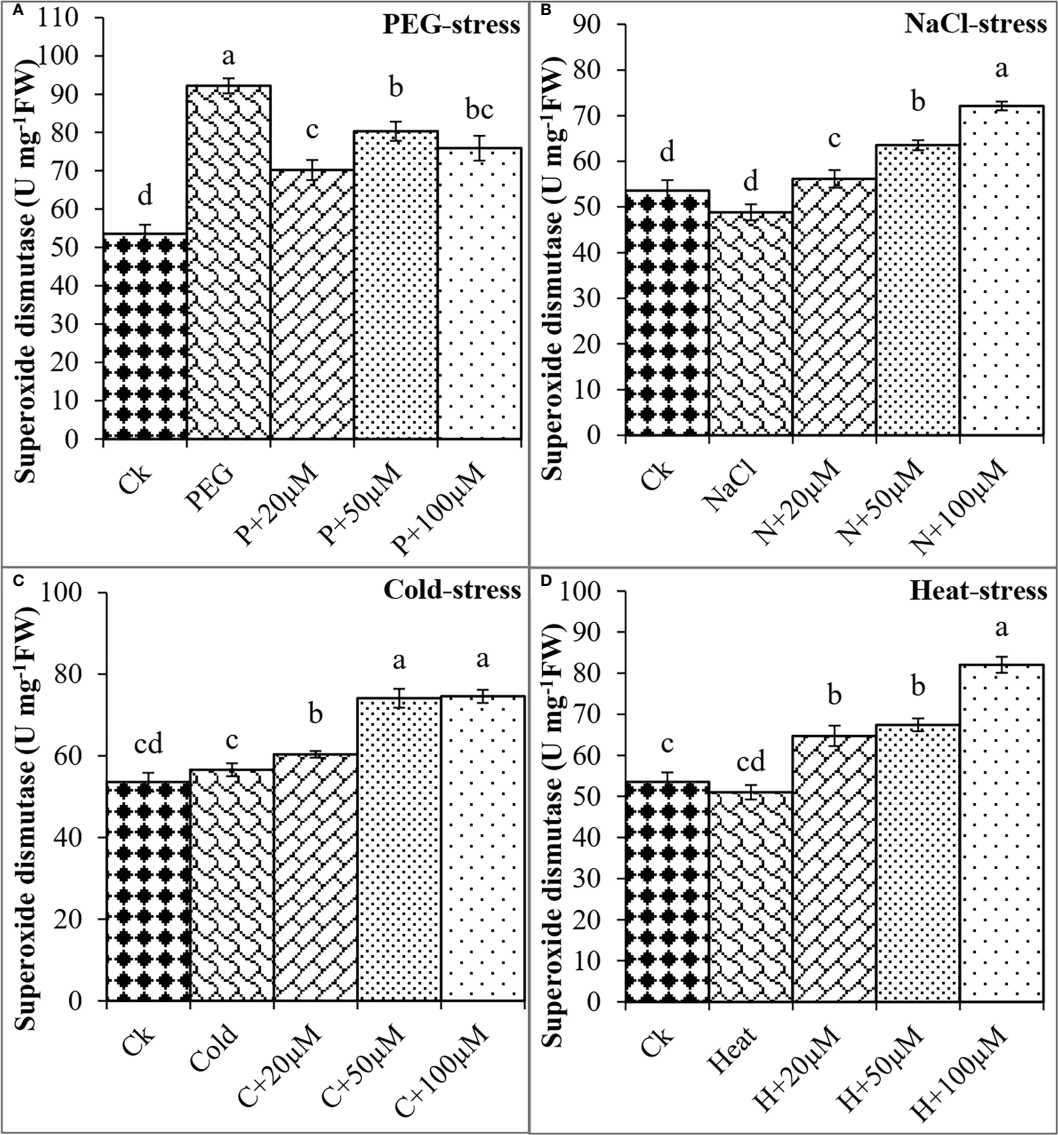
Effect of different concentrations of melatonin on the activity of superoxide dismutase under PEG **(A)**, NaCl **(B)**, Cold **(C)**, and Heat **(D)**. Values are the means ± SD (n=3). Different letters on the bars show a statistical significance level at *p<0.05*. Here, µM is indicating (µmole L^-1^).

**Figure 7:**
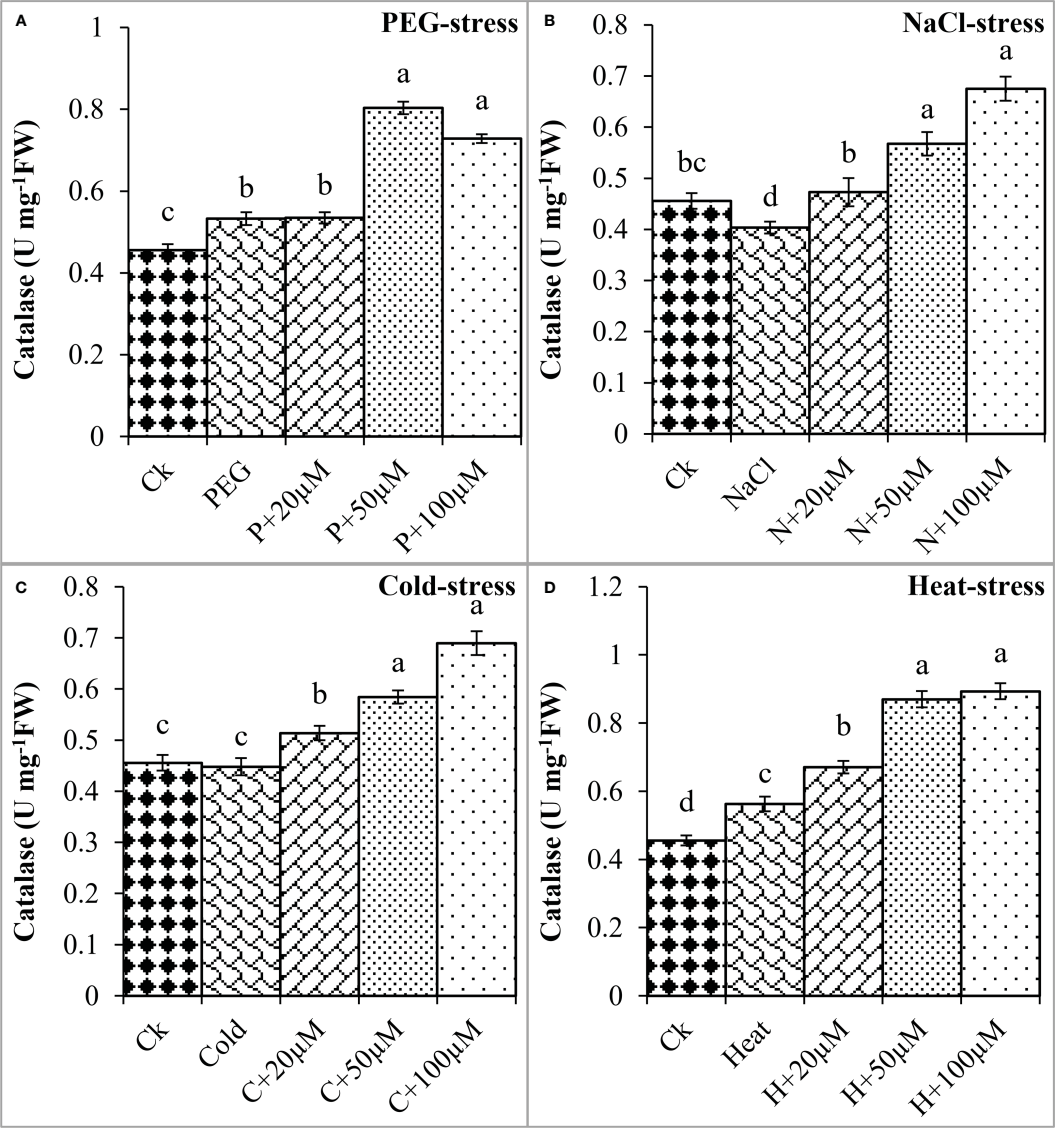
Effect of different concentrations of melatonin on the activity of Catalase (CAT) under PEG **(A)**, NaCl **(B)**, Cold **(C)**, and Heat **(D)**. Values are the means ± SD (n=3). Different letters on the bars show a statistical significance level at *p<0.05*. Here, µM is indicating (µmole L^-1^).

In addition, the activities of antioxidant enzymes including SOD, CAT, POD, and APX were found negatively regulated by PEG, NaCl, Cold, and Heat stresses whereas melatonin positively impacted these activities to reduce the effects of oxidative damage. PEG and Cold increased the SOD by 72% and 6% as compared to control; however, melatonin at different levels progressively improved the SOD activity under PEG and Cold (**Figure 6**). Moreover, NaCl and Heat decreased the SOD activity by 9% and 5% over the control. In contrast, melatonin at 20 μmol L^-1^, 50 μmol L^-1^, and 100 μmol L^-1^ increased the SOD by 14%, 30%, and 47% over NaCl (**Figure 6B**), and 27%, 32% and 60% over Heat stress (**Figure 6D**) respectively. Furthermore, CAT activity was found to improve due to melatonin under all these stresses (**Figure 7**).

**Figure 8:**
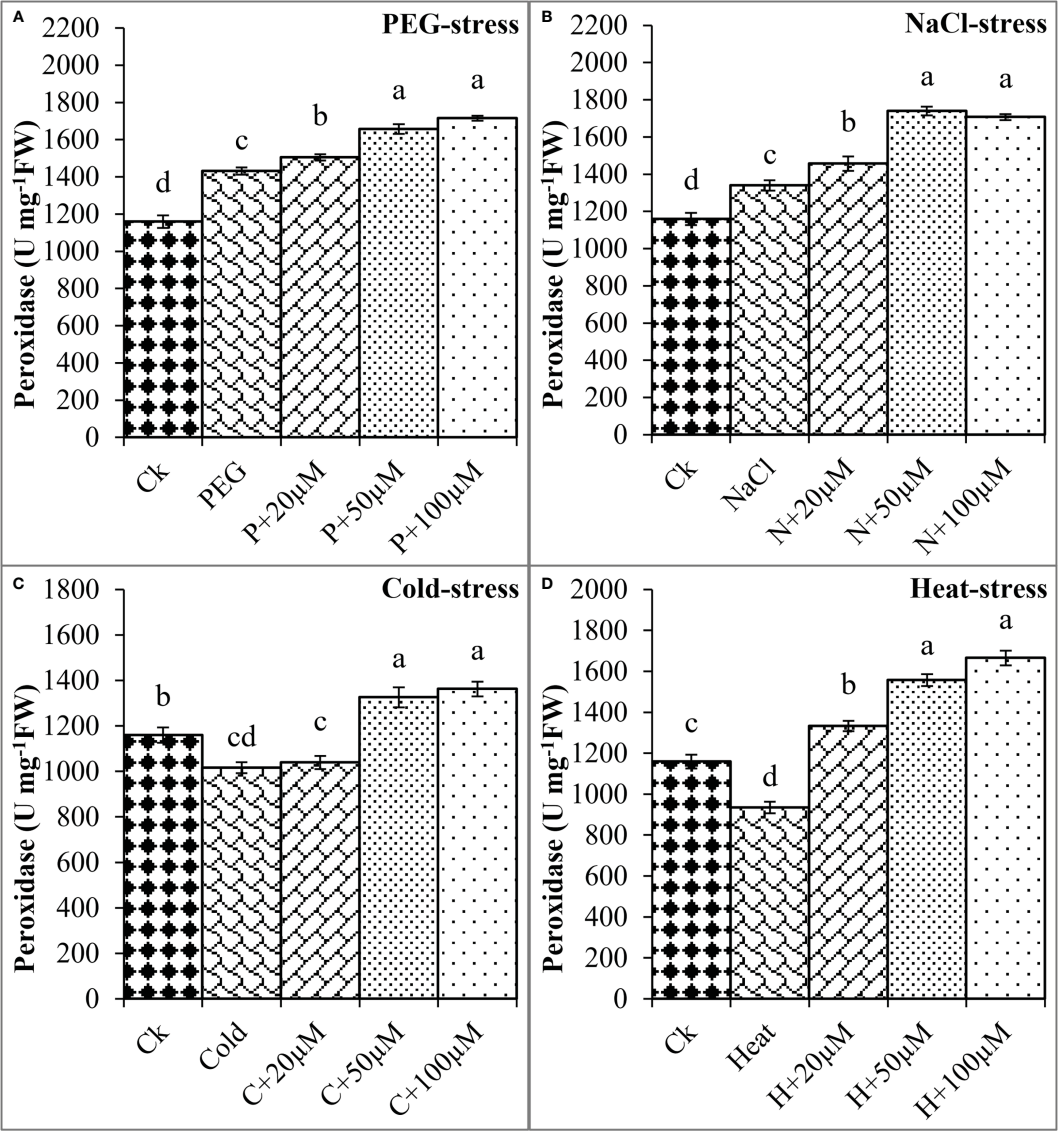
Effect of different concentrations of melatonin on the activity of Peroxidase (POD) under PEG **(A)**, NaCl **(B)**, Cold **(C)**, and Heat **(D)**. Values are the means ± SD (n=3). Different letters on the bars show a statistical significance level at *p<0.05*. Here, µM is indicating (µmole L^-1^).

**Figure 9:**
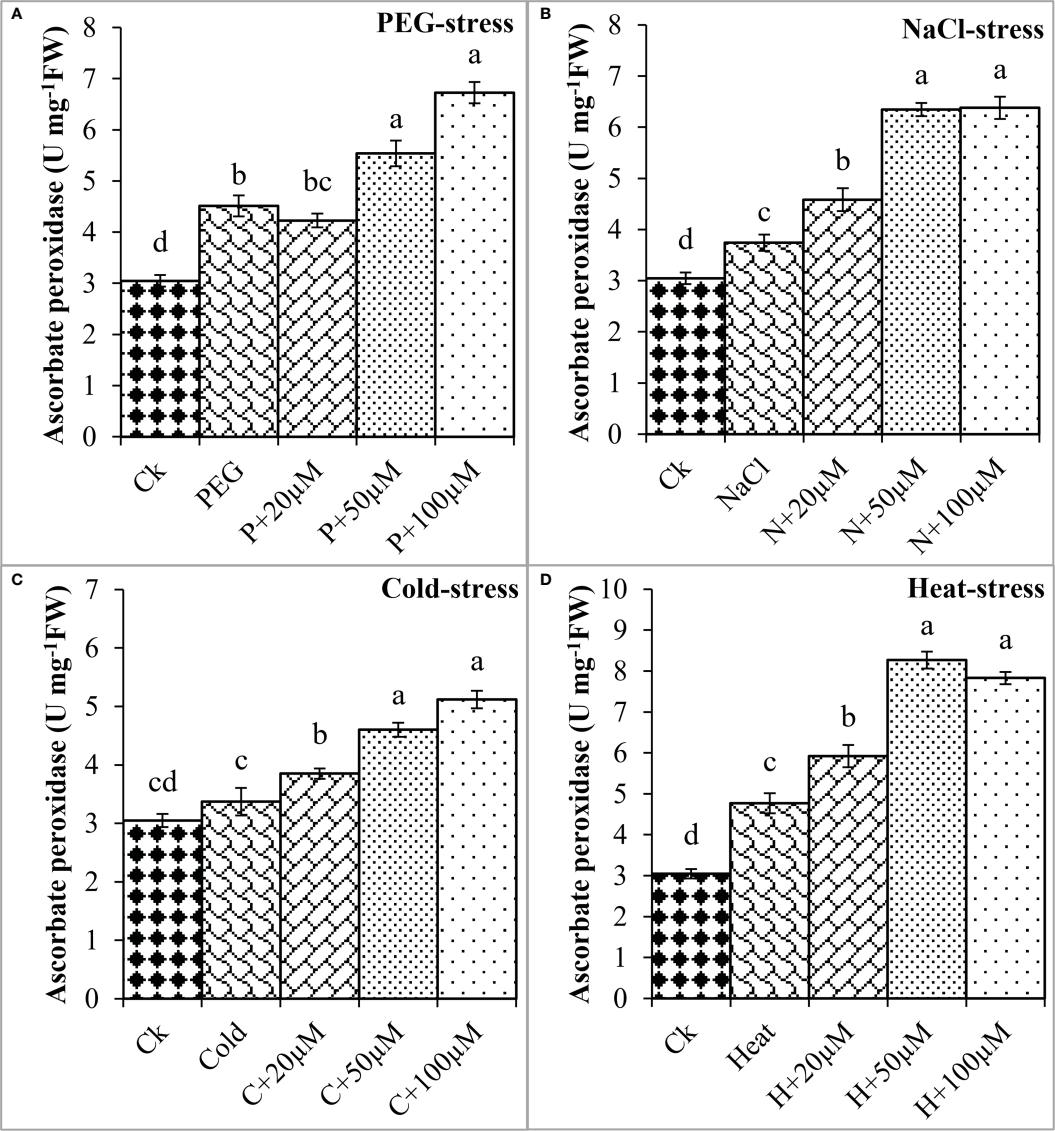
Effect of different concentrations of melatonin on the activity of Ascorbate peroxidase (APX) under PEG **(A)**, NaCl **(B)**, Cold **(C)**, and Heat **(D)**. Values are the means ± SD (n=3). Different letters on the bars show a statistical significance level at *p<0.05*. Here, µM is indicating (µmole L^-1^).

The melatonin at 20 μmol L^-1^, 50 μmol L^-1^, and 100 μmol L^-1^ enhanced the CAT activity by 0.3%, 50%, and 36% over PEG, 17%, 40%, and 67% over NaCl, 14%, 30%, and 54% over Cold, and 19%, 54%, and 58% over Heat. In the same line, the POD activity was differentially regulated by PEG, NaCl, Cold, and Heat stress however, progressively increased due to different concentrations of melatonin under all the stresses (**Figure 8**). PEG and cold decreased the POD activity whereas; NaCl and Heat increased the POD activity as compared to the control. Besides, melatonin at 20 μmol L^-1^, 50 μmol L^-1^, and 100 μmol L^-1^ increased the POD activity by 5%, 15%, and 19% over PEG (**Figure 8A**), 8%, 30%, and 27% over NaCl (**Figure 8B**), 2%, 30%, and 34% over Cold (**Figure 8C**), and 42%, 67%, and 78% over Heat (**Figure 8D**). In addition, the APX activity was also found to increase due to PEG, NaCl, Cold, and Heat stresses, and different levels of melatonin further boosted the APX activity under these stresses (**Figure 9**). Melatonin at 20 μmol L^-1^, increased the APX activity by 22%, 14%, and 24% over the NaCl, cold, and heat stresses respectively. Furthermore, 50 μmol L^-1^ increased the APX by 22%, 69%, 36%, and 73%, and 100 μmol L^-1^ by 49%, 70%, 51%, and 64% over the PEG, NaCl, Cold, and Heat, respectively. Overall, the melatonin at all levels improved the activities of SOD, CAT, POD, and APX but 50 μmol L^-1^ and 100 μmol L^-1^ significantly impacted antioxidant enzymes activities under these stresses.

**Figure 10:**
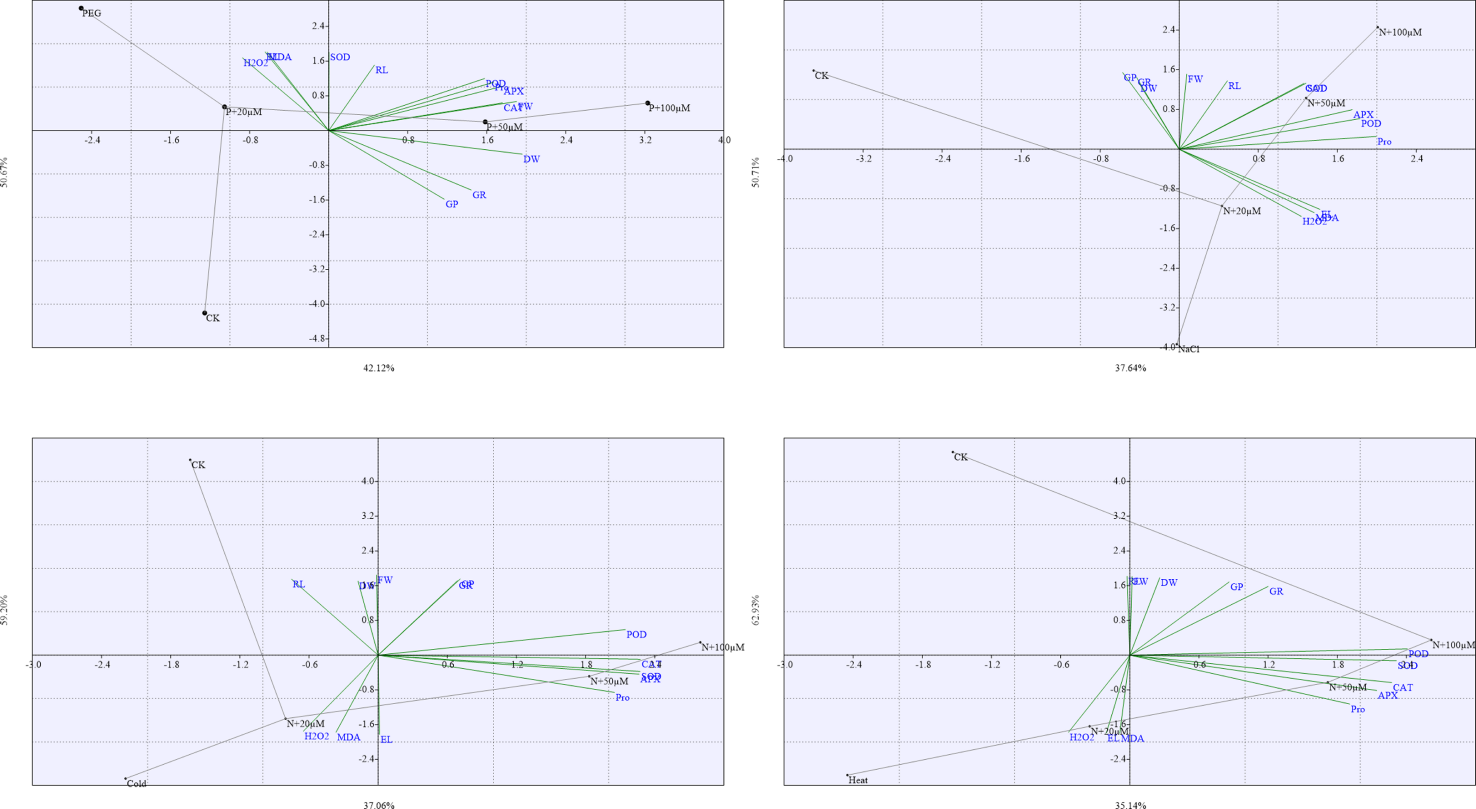
The Loading plots of principal component analysis (PCA) of different measured parameters of soybean seed germination at different concentrations of melatonin under drought **(A)**, salt **(B)**, cold **(C)**, and heat **(D)** stress. The abbreviations used in the plots are as follows; germination potential (GP), germination rate (GR), fresh weight (FW), dresh weight (DW), proline (Pro), electrolyte leakage (EL), malondialdehyde (MDA), hydrogen peroxide (H_2_O_2_) superoxide dismutase (SOD), catalase (CAT), peroxidase (POD), and ascorbate peroxidase (APX). Here, µM is indicating (µmole L^-1^).

The loading plots of principal component analysis (PCA) evaluated the impact of drought, salt, cold, and heat stresses and exogenous melatonin on different parameters of soybean seeds during germination (**Figure 10**). Among all the components, the two components i.e. component 1 and component 2 covered almost 90-95% of whole dataset that made the largest portion of all components. The component 1 distributed about 50.67% and component 2 distributed about 42.12% (**Figure 10A**), component 1 distributed about 50.71% and component 2 distributed about 37.64% (**Figure 10B**), component 1 distributed about 59.20% and component 2 distributed about 37.06% (**Figure 10C**), and component 1 distributed about 62.93% and component 2 distributed about 35.14% (**Figure 10D**), of whole dataset. All the measured parameters were dispersed in the dataset of these two components.

## Discussion

Soybean is an important leguminous crop and a major source of plant protein for humans, and animals intake it as an essential grain, oil, and feed (Yang et al., 2018b). Global production of soybean is facing major challenges to meet the increasing demand of the world’s population. Climatic changes are critical and devastating factors to decrease the growth and production of plants (Chaudhry and Sidhu, 2022). The most sensitive and crucial stage of plant growth is seed germination (Patanè et al., 2016). However, abiotic stresses including drought, salt, cold, heat, and heavy metals negatively affect the germination of seeds by influencing the internal metabolic and physiological processes (Sethy and Ghosh, 2013; Cao et al., 2019). This is because a stressful environment leads to reduce water intake and energy supply to seeds, negative regulation of ROS, osmoprotectants, antioxidant defense system, and hormonal balance (Shu et al., 2018; Sharma and Zheng, 2019). In the same context, the present study suggested that 15% PEG, 150mM NaCl, 10°C cold, and 30°C heat strongly inhibited the germination of soybean seeds.

Besides, different plant growth regulators have been widely used to treat seeds before sowing to improve resistance against abiotic stresses and promote germination (Liang et al., 2018; Bai et al., 2020). Among them, melatonin is being widely used to enhance growth and other physiological attributes of different crops and showing remarkable results under normal stressful conditions (Wang et al., 2018). Naturally, melatonin is synthesized by L-tryptophane which is an essential amino acid and is also used for protein biosynthesis (Arnao and Hernández-Ruiz, 2014). Therefore, under normal and stressful conditions proteins stabilize the various metabolic functions of cells including plants and animals. Previous studies have reported positive and beneficial effects of melatonin under normal and stressful conditions in different plants (Sharma and Zheng, 2019). Our study depicted that lower concentrations of melatonin remarkably improved the germination potential (GP), germination rate (GR), radical length (RL), fresh weight (FW), and dry weight (DW) however; higher concentrations did not show remarkable improvements under abiotic stress conditions. Previous studies revealed that melatonin at 100 µM improved the germination indices of cotton (*Gossypium hirsutum* L.) and wheat (*Triticum aestivum* L.) under PEG and heat stress (Bai et al., 2020; Iqbal et al., 2021), 20 µM under salt stress (Chen et al., 2020), and 5-20 µM under cold stress in *Stevia rebaudiana* (Simlat et al., 2018). It is suggested that melatonin crosstalk with ABA and GA, helps in breaking seed dormancy, facilitates more water uptake, and activates secondary messengers to enhance GP, GR, and GI, and the successful establishment of seedlings under abiotic stresses (Zhang et al., 2014a; Xiao et al., 2019). Furthermore, the higher concentrations of melatonin are not beneficial at all because sometimes it becomes the cause of growth inhibition that depends upon the type of species and duration of melatonin treatment (Cao et al., 2019). Thus, the above presented literature is in line with the findings of the present study.

Generally, PEG, NaCl, cold, and heat lead to the generation of excessive production of ROS, and MDA content as lipid peroxidation, and increase the electrolyte leakage which results in the decrease of germination and early growth of seedlings (Chen et al., 2020; Awan et al., 2021). However, melatonin has been reported to counteract H_2_O_2_ and reduce MDA content and EL in response to abiotic stresses (Wang et al., 2018; Seleiman et al., 2021). For example, melatonin inhibited the excessive production of ROS and MDA, and deceased the EL that cause oxidative damage in tomato (*Solanum lycopersicum* L.) and maize (*Zea mays* L.) under chilling stress (Liu et al., 2015; Cao et al., 2019), in tomato (*Solanum lycopersicum* L.) under heat stress (Jahan et al., 2019), in cotton (*Gossypium hirsutum* L.) under drought and salt stresses (Bai et al., 2020; Chen et al., 2020). It has been reported that H_2_O_2_ is associated with embryo elongation during seed germination and melatonin effectively and positively regulates its content directly or indirectly inside the cells (Zhang et al., 2014b; Bai et al., 2020). Basically, at the beginning of seed germination, a series of physiological processes take place including absorption of water, activation of various enzymes and hormones, membrane repair activities, and degradation of storage substances inside the seeds (Hegarty, 1978; Ma et al., 2017). So, abiotic stresses lead to inhibition or slow down these mechanisms and cause oxidative damage whereas melatonin positively regulates internal homeostasis by reducing secondary stresses caused by drought, salt, chilling and heat (Bai et al., 2020; Cao et al., 2022). Similarly, our results showed that all four stresses dramatically increased the H_2_O_2_, MDA, and EL whereas; melatonin at 50 μmol L^-1^ and 100 μmol L^-1^ considerably decreased the H_2_O_2_, MDA content, and EL under the drought, salt, cold, and heat stresses in soybean which are consistent with previous findings as in tomato (*Solanum lycopersicum* L.), wheat (*Triticum aestivum* L.), chickpea (*Cicer arietinum* L.), pepper (*Capsicum annuum* L.), rice (*Oryza sativa* L.), and cotton (*Gossypium hirsutum* L.) (Cao et al., 2019; Jahan et al., 2019; Bai et al., 2020; Buttar et al., 2020; Korkmaz et al., 2021; Raza et al., 2022) Based on these observations, an optimal level of melatonin could effectively reduce the overproduction of ROS, MDA, and decrease EL in terms of oxidative damage and provide protection by activating defensive system; however, this phenomenon might be dose-, time duration-, stage-, and species-dependent (Raza et al., 2022).

The accumulation of protective substances/osmoprotectants plays a crucial role in maintaining internal stability and protecting cells from abiotic stress damage (Bai et al., 2020). Melatonin has been reported to increase the accumulation of proline in response to drought (Zhang et al., 2020), salt (Chen et al., 2020), cold (Cao et al., 2019), and heat stress (Imran et al., 2021), and enhanced the germination and early seedlings growth (Bai et al., 2020). The present study showed that abiotic stresses accelerated the level of proline whereas; the application of melatonin progressively increased the level of proline during seed germination under these stresses. These results suggested that melatonin can positively regulate the osmotic substances inside the cells and enhance the drought, salt, cold, and heat stress tolerance. Pre-treatment of melatonin at lower concentrations of 1-100 µM increased proline in cotton under drought (Bai et al., 2020), in tomato under salt (Siddiqui et al., 2019), in maize under chilling (Cao et al., 2019), and in brassica (*Brassica napus* L.) under Se-stress, and in strawberry (*Fragaria* × *ananassa*) under heat stress (Ulhassan et al., 2019). Mainly, proline is involved in ROS detoxification, protecting membrane integrity, cell organelles, and osmotic adjustments (Hosseinifard et al., 2022). Thus, accumulation of proline *via* pre-treatment of melatonin could increase resistance against abiotic stresses.

Furthermore, plants possess a complex defensive mechanism to inhibit the excessive production of ROS and reduce oxidative damage (Ahammed et al., 2020; Bai et al., 2020; Khan et al., 2023). This defensive system is composed of different antioxidant enzymes including SOD, CAT, POD, and APX that perform a variety of defensive functions (Khan et al., 2020). SOD is considered the first line of defense that can convert O^2-^ to H_2_O_2_, which is further converted to water and oxygen through CAT (Li et al., 2017; Rizwan et al., 2019). The SOD and POD are important antioxidants that can readily eliminate ROS from cells and protect cell membrane stability from lipid peroxidation (Xiao et al., 2019). Pre-treatment of melatonin has been reported to activate the defense system by increasing the activities of antioxidant enzymes that scavenge the radicals and minimize the oxidative damage in response to stressful conditions (Zhang et al., 2014a; Sharif et al., 2018; Bai et al., 2020; Mushtaq et al., 2022). In the present study, drought and heat stress decreased the activities of antioxidant enzymes which might be due to overproduction of ROS and strong oxidative damage as found in previous studies (Zeng et al., 2022). Moreover, pre-treatment of melatonin progressively enhanced the activities of antioxidant enzymes and maximum enhancement was observed at MT2 and MT3 in response to all stresses. These findings suggest that melatonin increases the tolerance of soybean seeds by reducing oxidative damage under drought, salt, cold, and heat stresses. Our findings are consistent with previous studies that reported the pre-treatment of melatonin at 1-100 µM improved the activities of SOD, CAT, POD, and APX in cotton under drought and salt stress (Bai et al., 2020; Chen et al., 2020), alfalfa (*Medicao sativa* L.), barley (*Hordeum Vulage* L.*)*, and corn under cold stress (Li et al., 2016; Cao et al., 2019; Irshad et al., 2021), wheat and strawberry under heat stress (Buttar et al., 2020; Manafi et al., 2022). Pieces of evidence revealed that melatonin can enter the seeds through the seed coat as a result of seed priming and regulate the various physiological mechanisms inside the seed including, activation of internal hormonal signaling, stimulation of secondary metabolites, and regulate the sugar metabolism that is responsible for early germination under normal and stressful condition (Bai et al., 2020; Zhang et al., 2020). Furthermore, melatonin has been reported to positively regulate the internal ROS level of seed which is crucial to stimulate the breakage of seed dormancy and resulting in seed germination under stressful conditions (Arnao and Hernández-Ruiz, 2014; Bai et al., 2020). Based on the previous literature and findings of the present study, it is suggested that seed pre-treatment with melatonin can efficiently promote the seed germination of soybean and can potentially alleviate the deleterious effects of abiotic stresses including drought, salt, cold, and heat. Lower concentrations of melatonin at MT1, MT2, and MT3 perform significant functions in improving the germination, proline accumulation, and activities of antioxidant enzymes, and reducing the oxidative damage in terms of ROS, MDA, and EL as compared to higher concentrations under stressful condition. Similarly, present study depicted that seed priming with melatonin reduced oxidative stress in terms of H_2_O_2_, MDA, and EL, and enhanced the activities of SOD, CAT, POD, and APX under different abiotic stresses which promoted the early germination and improved the germination responses in soybean. In addition, the effects of melatonin on seed germination, early seedlings growth, and other physiological attributes are dependent on the type of abiotic stress, species, type of plants, growth stage, and duration of stresses (Raza et al., 2022). It can be concluded that melatonin shows variable positive effects under different types of stresses. Thus, further molecular and transcriptome studies would be required to explore the melatonin-mediated metabolic pathways and important genes that are responsible to enhance soybean tolerance against abiotic stresses during germination.

## Conclusion

Abiotic stresses including drought, salt, cold, and heat lead to an increase in the oxidative damage by increasing the H_2_O_2_, MDA, and EL which results in membrane damage and other secondary stresses in soybean seeds during germination. Pre-treatment of soybean seeds with melatonin differentially regulated and positively improved the germination indices and other physiological processes. Pre-treatment of melatonin at 20 μmol L^-1^, 50 μmol L^-1^, and 100 μmol L^-1^ was found significant in improving germination potential, germination rate, radical length, fresh weight, and dry weight of soybean seed germination however, remarkable alleviation of abiotic stresses was noticed at 50 μmol L^-1^ and 100 μmol L^-1^. Both these levels significantly increased the activities of antioxidant enzymes such as SOD, CAT, POD, and APX, osmoprotectant (proline), and decreased oxidative damage under abiotic stresses in soybean seed germination. The present study suggests that these specific concentrations of melatonin (50 μmol L^-1^ and 100 μmol L^-1^) can positively enhance seed germination and improve tolerance against the given level of drought, salt, cold, and heat stresses. Furthermore, molecular bases with detailed regulatory networks of physiological and biochemical mechanisms in the seeds of different crop plants are needed to be further explored before the field implication. Besides, this study provides the valuable bases for the protective and successful germination of soybean with improved tolerance against abiotic stresses however; future studies are required to deeply understand the melatonin-mediated stress tolerance mechanisms in different crops during germination.

## Data availability statement

The original contributions presented in the study are included in the article/supplementary material. Further inquiries can be directed to the corresponding author.

## Author contributions

FY supervised the project. SA and IK performed the experiment and analyzed the data. SA and IK participated in the writing of manuscript. JG, XT, QW, and FY contributed in revising and editing the manuscript. All authors contributed to the article and approved the submitted version.
